# Supplementary material for: HIVprotI: an integrated web based platform for prediction and design of HIV proteins inhibitors
Source: J Cheminform. 2018 Mar 9;10:12. doi: 10.1186/s13321-018-0266-y (PMC5845081; doi:10.1186/s13321-018-0266-y)
Supplement: Supplementary file 2 — Additional file 2. Source code of HIVProtI web server. [file 13321_2018_266_MOESM2_ESM.zip › HIVprotI_Source-code/hivprotI/weka-3-6-6/doc/weka/classifiers/trees/m5/PreConstructedLinearModel.html]

PreConstructedLinearModel


---


|  |  |  |
| --- | --- | --- |
| **Overview** \| **Package** \| **Class** \| **Tree** \| **Deprecated** \| **Index** \| **Help** \| **Weka's home** | |  |
| **PREV CLASS**   **NEXT CLASS** | **FRAMES**    **NO FRAMES**     **All Classes** |  |
| SUMMARY: NESTED \| FIELD \| CONSTR \| METHOD | DETAIL: FIELD \| CONSTR \| METHOD |  |


---


## weka.classifiers.trees.m5 Class PreConstructedLinearModel

```
java.lang.Object
  weka.classifiers.Classifier
      weka.classifiers.trees.m5.PreConstructedLinearModel
```

**All Implemented Interfaces:**: java.io.Serializable, java.lang.Cloneable, CapabilitiesHandler, OptionHandler, RevisionHandler

---

``` public class PreConstructedLinearModel extends Classifier implements java.io.Serializable ```

This class encapsulates a linear regression function. It is a classifier
but does not learn the function itself, instead it is constructed with
coefficients and intercept obtained elsewhere. The buildClassifier method
must still be called however as this stores a copy of the training data's
header for use in printing the model to the console.

**Version:**
:   $Revision: 1.6 $

**Author:**
:   Mark Hall (mhall@cs.waikato.ac.nz)

**See Also:**: Serialized Form

---

**Constructor Summary**

`PreConstructedLinearModel(double[] coeffs,
double intercept)`
  
          Constructor


| **Method Summary** | |
| --- | --- |
| `void` | `buildClassifier(Instances instances)`             Builds the classifier. |
| `double` | `classifyInstance(Instance inst)`             Predicts the class of the supplied instance using the linear model. |
| `double[]` | `coefficients()`             Return the array of coefficients |
| `java.lang.String` | `getRevision()`             Returns the revision string. |
| `double` | `intercept()`             Return the intercept |
| `int` | `numParameters()`             Return the number of parameters (coefficients) in the linear model |
| `java.lang.String` | `toString()`             Returns a textual description of this linear model |

**Methods inherited from class weka.classifiers.Classifier**

`debugTipText, distributionForInstance, forName, getCapabilities, getDebug, getOptions, listOptions, makeCopies, makeCopy, setDebug, setOptions`

**Methods inherited from class java.lang.Object**

`equals, getClass, hashCode, notify, notifyAll, wait, wait, wait`

**Constructor Detail**

### PreConstructedLinearModel

```
public PreConstructedLinearModel(double[] coeffs,
                                 double intercept)
```

:   Constructor

    **Parameters:**: `coeffs` - an array of coefficients: `intercept` - the intercept


**Method Detail**

### buildClassifier

```
public void buildClassifier(Instances instances)
                     throws java.lang.Exception
```

:   Builds the classifier. In this case all that is done is that a
    copy of the training instances header is saved.

    :   **Specified by:**: `buildClassifier` in class `Classifier`
    :   **Parameters:**: `instances` - an `Instances` value **Throws:**: `java.lang.Exception` - if an error occurs

---


### classifyInstance

```
public double classifyInstance(Instance inst)
                        throws java.lang.Exception
```

:   Predicts the class of the supplied instance using the linear model.

    :   **Overrides:**: `classifyInstance` in class `Classifier`
    :   **Parameters:**: `inst` - the instance to make a prediction for **Returns:**: the prediction **Throws:**: `java.lang.Exception` - if an error occurs

---


### numParameters

```
public int numParameters()
```

:   Return the number of parameters (coefficients) in the linear model

    :   **Returns:**: the number of parameters

---


### coefficients

```
public double[] coefficients()
```

:   Return the array of coefficients

    :   **Returns:**: the coefficients

---


### intercept

```
public double intercept()
```

:   Return the intercept

    :   **Returns:**: the intercept

---


### toString

```
public java.lang.String toString()
```

:   Returns a textual description of this linear model

    :   **Overrides:**: `toString` in class `java.lang.Object`
    :   **Returns:**: String containing a description of this linear model

---


### getRevision

```
public java.lang.String getRevision()
```

:   Returns the revision string.

    :   **Specified by:**: `getRevision` in interface `RevisionHandler` **Overrides:**: `getRevision` in class `Classifier`
    :   **Returns:**: the revision


---


|  |  |  |
| --- | --- | --- |
| **Overview** \| **Package** \| **Class** \| **Tree** \| **Deprecated** \| **Index** \| **Help** \| **Weka's home** | |  |
| **PREV CLASS**   **NEXT CLASS** | **FRAMES**    **NO FRAMES**     **All Classes** |  |
| SUMMARY: NESTED \| FIELD \| CONSTR \| METHOD | DETAIL: FIELD \| CONSTR \| METHOD |  |


---
